# Glucocorticoids modify intracranial pressure in freely moving rats

**DOI:** 10.1186/s12987-023-00439-y

**Published:** 2023-05-25

**Authors:** Connar Stanley James Westgate, Ida Marchen Egerod Israelsen, Christina Kamp-Jensen, Rigmor Højland Jensen, Sajedeh Eftekhari

**Affiliations:** Danish Headache Center, Dept of Neurology, Rigshospitalet-Glostrup, Glostrup Research Institute, University of Copenhagen, Glostrup, Denmark

**Keywords:** Intracranial pressure, Glucocorticoid, Prednisolone, Corticosterone, Choroid plexus

## Abstract

**Background:**

Glucocorticoids (GCs) are widely prescribed for a variety of inflammatory diseases, but they are also used to treat raised intracranial pressure (ICP) caused by trauma or oedema. However, it is unclear if GCs independently modulate ICP and if GCs are involved in normal ICP regulation. In this study, we aimed to assess the ICP modulatory effects of GCs and their molecular consequences on choroid plexus (CP).

**Methods:**

Adult female rats were implanted with telemetric ICP probes for physiological, continuous ICP recordings in a freely moving setup. Rats received prednisolone or vehicle via oral gavage in a randomized acute (24 h) ICP study. In a subsequent study rats received corticosterone or vehicle in drinking water for a 4-week chronic ICP study. CP were removed, and the expression of genes associated with cerebrospinal fluid secretion were assessed.

**Results:**

A single prednisolone dose reduced ICP by up to 48% (P < 0.0001), where ICP was reduced within 7 h and was maintained for at least 14 h. Prednisolone increases ICP spiking (P = 0.0075) while not altering ICP waveforms. Chronic corticosterone reduces ICP by up to 44%, where ICP was lower for the entirety of the 4-week ICP recording period (P = 0.0064). ICP daily periodicity was not altered by corticosterone. Corticosterone ICP reduction was not accompanied by ICP spike differences or alteration in ICP spike periodicity. Chronic corticosterone treatment had modest effects on CP gene expression, lowering the expression of *Car2* at CP (P = 0.047).

**Conclusions:**

GCs reduce ICP in both the acute and chronic setting to a similar degree. Moreover, GCs did not modify the diurnal rhythm of ICP, suggesting the diurnal variation of ICP periodicity is not under explicit control of GCs. ICP disturbances should be considered a consequence of GC therapy. Based on these experiments, GCs may have broader ICP therapeutic uses, but side effects must be taken into consideration.

**Supplementary Information:**

The online version contains supplementary material available at 10.1186/s12987-023-00439-y.

## Background

Glucocorticoids (GCs) are highly prescribed drugs and are used as a treatment in multiple disorders such as rheumatoid arthritis, asthma, multiple sclerosis, allergies and inflammatory bowel diseases [1–3]. GCs are also an endogenous hormone class that is essential for life [4]. Therapeutically, GCs are associated with strong initial therapeutic benefit. However, chronic therapy confers a host of side effects including: hypothalamic-pituitary-adrenal axis dysfunction, osteoporosis, cachexia, promotion of central obesity and impaired wound healing amongst others [1]. GCs elicit a multitude of anti-inflammatory and catabolic processes mediated through the GC activating enzyme 11β-hydroxysteroid dehydrogenase type 1 (11β-HSD1) converting cortisone (dehydrocorticosterone in rodents) to cortisol (corticosterone (CORT) in rodents) [5, 6]. These active GCs subsequently agonize the glucocorticoid receptor (GR), a transcription factor, which is ubiquitously expressed in tissues [5].

Another therapeutic use of GCs is treatment of cerebral oedema following traumatic brain injury, stroke and brain tumors on the observation they reduce oedemic processes, thus reducing intracranial pressure (ICP) according to the Monro-Kellie hypothesis [7–10]. Although GCs have been demonstrated to reduce ICP in an experimental model of raised ICP, it is currently unknown if GCs can reduce ICP independently of their anti-inflammatory properties, i.e. through modulating cerebrospinal fluid (CSF) dynamics [11]. Moreover, given that GC therapy is often chronic, it is unknown what effect chronic administration of GCs has on normal ICP.

The potential mechanism underlying a GC mediated modulation of ICP remains controversial. It is clear that GCs can reduce oedemic processes, however, the effects of GCs on ICP dynamics are unclear. Although it has been demonstrated that choroid plexus (CP), the organ that secretes the majority of the CSF, expresses both glucocorticoid receptor (GR) and 11β-HSD1, the effect of GCs on CSF secretion is debated with different studies reporting opposite responses [12–14]. Moreover, GCs are reported to have no effect on CSF drainage [15, 16]. Clinical studies have also suggested that excess GCs could be involved in raised ICP disorders, such as in Idiopathic intracranial hypertension (IIH) and iatrogenic pseudotumour cerebri syndrome [17–20].

Given the lack of clear experimental evidence for the direct effect of therapeutic GCs on ICP, we aimed to investigate the effect of therapeutic doses of GCs on ICP in acute and chronic dosing regimens.

## Methods

### Animals

A total of 22 female adult Wistar rats (Charles River, Denmark) and 4 Sprague-Dawley rats (Taconic, Denmark) at 10 weeks of age were used. This study used only female rats due to the potential pathophysiological link to IIH that affects mainly females. The rats were housed in the animal facility at Research Institute, Rigshospitalet-Glostrup, Denmark, and allowed to acclimatize for at least 1 week after arrival prior to procedures. Rats were kept in an inverted light dark cycle (12 h:12 h, where it was dark for rats when daytime for investigators), with ad libitum access to standard rodent diet and water. All experiments were approved by the Institutional Animal Care and Use Committee, the Danish Animal Experiments Inspectorate (2019-15-0201-00365) and comply with ARRIVE guidelines.

#### ICP surgery

To record ICP, fully implantable factory calibrated telemeters (Kaha Sciences Ltd, Auckland, New Zealand) were used as previously described [21]. On the day of surgery, anesthesia was induced with an intraperitoneal injection of ketamine (100 mg/kg) and xylazine (5 mg/kg), anesthesia was deemed to be induced when the absence of pedal and blink reflexes were observed. Anesthesia was maintained with 1-third of a full dose of ketamine/xylazine when pedal or blink reflexes were observed. In brief, the telemeter body was implanted in the abdominal cavity of the rats, thereafter the connective catheter was tunneled in underneath the skin up to the parietal bone. The rats were then placed in a stereotactic frame to securely place the sensor tip epidurally. Epidural ICP is identical to measuring ICP intraventricularly in rats [22]. After surgery rats were single-housed for 7–10 days to recover, where they received post-surgical treatment the first two days following ICP surgery. The post-surgical treatments consisted of subcutaneous injections with 2 ml saline, 5 mg/kg carprofen (Rimadyl, Pfizer), 0.03 mg/kg buprenorphine (Temgesic, Pharmaceuticals limited, UK) and 10 mg/kg enrofloxacin (Baytril, Bayer). The rats also had access to 0.4 mg/kg buprenorphine mixed in Nutella (Ferrero, Germany) for two days. Rats were co-housed one week after surgery and throughout the rest of recording period.

#### ICP recordings

Prior to surgery, telemeters were offset test to determine the zero point of an individual telemeter as previously described [21]. ICP recordings started at the day of surgery and continued until sacrifice, where rats were placed upon Kaha Science Smartpads in normal cages. For data acquisition from Smartpad, recording and display, PowerLab and LabChart software (v8.0, ADInstruments, New Zealand) were used. The telemeters sampled ICP at 2 kHz, where the Smartpad lowpass filtered at 1 kHz gave a final sampling frequency of 1 kHz. For the chronic dosing of corticosterone ICP was recorded for 28 days after implantation. Calculation of ICP was done as previously described [21]. We have previously demonstrated that ICP increases following surgery to a stable level over 9 days [21]. Consequently, acute treatments were given after the rats ICP had recovered from surgery. The ICP is presented as daily mean ICP, calculated from the average of every hour. For the acute dosing of prednisolone ICP was measured for 24 h after administration and presented as Δ change in ICP from baseline. All ICP analysis was blinded to treatment group. We have previously demonstrated that ICP telemeter drift is minimal with the present setup [21].

#### Administration of prednisolone by oral gavage for acute ICP recordings

To assess the effects of prednisolone (PRED) on ICP, PRED tablets (EQL Pharma AB, Lund, Sweden) were dissolved in isotonic saline and administered via oral gavage. The rats were acclimatised to the gavage procedure for a week prior to ICP surgery to reduce stress associated with the procedure. The dose of PRED was determined by reversing the FDA first in man equation (rat dose (mg/kg) = human dose (mg/kg)*6.2), where a pulse dose of PRED is between 10 and 20 mg/kg in humans, equating to between 62 and 124 mg/kg in a rat [23]. We decided to administer ~ 100 mg/kg in 1 ml of isotonic saline. 1 ml of isotonic saline was used as a vehicle control. Rats received the drugs in a sequential study, vehicle first and PRED second, with a 3-day washout period between administration. Treatments were given after the rats ICP had recovered from surgery and were administered when rats were in their active phase, i.e. when the housing room was dark. After completion of the acute study rats were sacrificed using I.P pentobarbitone/lidocaine.

#### Administration of corticosterone in drinking water for chronic ICP recordings

Corticosterone (CORT) (27,840 Sigma-Aldrich, St. Louis, MI) was administered in the drinking water (400 mg/L) dissolved in 2.5% ethanol. The vehicle group received water containing 2.5% ethanol. 400 mg/L has been shown to be sufficient to reduce serum CORT levels, thus indicating that the dose given is in excess to physiological levels, acting as a replacement to adrenal CORT [24]. The water bottles were made of opaque glass to prevent photodegradation of CORT. Rats were given CORT water or vehicle water for 2 weeks prior to ICP surgery as a pretreatment. Treatment of the drinking water continued immediately after surgeries, for 4 more weeks prior to sacrifice using I.P pentobarbitone/lidocaine. No impaired wound healing or adverse events were observed in the CORT treated group after surgery. CORT administration in water was selected to reduce chronic handling stress and minimize the ethanol exposure due to the insolubility of other corticosteroids.

#### ICP spike analysis

For ICP spike analysis, LabChart was used. Here, custom detection settings were chosen in the cyclic measurement’s settings: normalisation with a 20 s window, noise set at 1mmHg, Minimum peak height of 2.9282 standard deviations, detection parameters two-sided height with a minimum period of 250 ms, trigger of maximum and a peak search window of 30 s. With 10ms smoothing, all other settings as default. For all spike analysis, 1 h ICP spike bins were used. For the acute ICP spike analysis, ICP spikes were assessed in the 24 h prior to and after oral gavage of either vehicle or PRED. For the chronic ICP spike analysis, 5 days of ICP spikes were collected for each animal in days 16,17,18,19, and 20. For a given hour the ICP spikes were averaged for each day for each rat.

#### ICP periodicity analysis

To determine if there was periodicity in either ICP spikes or mean daily ICP we compared temporal curves with two types of fit, null hypothesis simple line and a cosine wave defined as (Y = Amplitude*cos((2*pi*X/Wavelength) + PhaseShift)) where wavelength was constrained to 24 h. Where a trace fitted a cosine curve, it was deemed to have diurnal variation and thus periodicity [12].

#### ICP waveform analysis

The spectral analysis of the ICP waveform was carried out on a 5-min stable section of ICP, i.e. without movement artefacts. A fast Fourier transformation (FFT) was carried out on raw, unprocessed 1 kHz data. The inbuilt FFT function of LabChart was utilised where spectral power was obtained utilising the following settings: FFT size of 128 K with a 93.75% window overlap, with a Hann (cosine-bell) data window model. The spectra generated were an average of 23 FFT. For temporal spectrograms, spectral analysis was carried out on a baseline (the hour prior to oral gavage), 3,6,12 and 24 h after drug administration and values made relative to the baseline value.

#### ICP variance

To assess ICP variance, the mean ICP for each second on day 20 was extracted for each rat. ICP was then normalized to the mean daily ICP for the individual rat, where daily ICP mean was set as zero. ICP was then expressed as a frequency distribution as a percentage, where ICP was distributed in 0.25 mmHg wide bins.

#### Adrenal gland mass

Following chronic CORT or vehicle treatment, rats with ICP implants were sacrificed with a single injection of pentobarbital/lidocaine. Upon sacrifice both adrenal glands were dissected out, perirenal adipose removed and wet weight assessed within 5 min of removal.

#### mRNA expression in CP

Following chronic CORT or vehicle treatment, rats with ICP implants were sacrificed with a single injection of pentobarbital/lidocaine. Rats were sacrificed around the time the housing room turned dark, thus as they are rousing for the day. CP were dissected out and snap frozen prior to storage at − 80 °C. The Tri reagent method was used for RNA isolation. Samples were incubated for 15 min in 1 mL TRIzol (Invitrogen™, Waltham, MA, USA), followed by trituration, and isopropanol was used to precipitate RNA. Purified RNA was quantified by UV spectrophotometry on NanoDrop (Thermo Scientific). RNA was converted to cDNA using Applied Biosystems High-Capacity cDNA Reverse Transcription Kit (Applied Biosystems, Waltham, MA) according to the manufacturer’s instructions.10 µl, 9ng cDNA reactions were carried out in triplicate. Taqman Gene Expression Master Mix (Applied Biosystems) combined with a TaqMan primer/probe to access expression of the following target genes which have been implicated in CSF secretion at the CP (all from Applied Biosystems): *Aqp1* (Rn_00562834_m1), *Aqp4* (Rn_01401327_s1), *Slc12a2* (Rn_00582505_m1), *Slc4a5* (Rn_01420902_m1), *Slc4a10* (Rn_00710136_m1), *Car2* (Rn_01462065_m1), *Car3* (Rn_01461970_m1), *Atp1a1* (Rn_01533986_m1), *Atp1b1* (Rn_00565405_m1), *Fxyd1* (Rn_00581299_m1), and GC action: *Nr3c1* (Rn_01405582_m1), *Tsc22d3* (Rn_00580222_m1) and *Hsd11b1* Rn00567167_m1). Reactions were run on QuantStudio™ 6 Pro Real-Time PCR System, 384-well (Applied Biosystems). Expression of target genes was normalized to the mean expression of *Actb* (Rn_00667869_m1) and *B2m* (Rn_00560865_m1) calculated as ΔCT = CT (target gene) − CT (reference gene). The data is presented as geometric mean of fold change (2-ΔΔCT) where ΔΔCT was calculated as ΔΔCT = ΔCT (target gene) – mean ΔCT (reference genes) and statistics were performed on the ΔCT values.

#### Statistics

Graphpad prism (V9.1, Graphpad Software Inc, San Diego, CA) was utilised to perform statistical analysis on data, where data are presented as mean ± S.E.M unless otherwise stated. ‘N’ in all instances represents biological replicates per group. Appropriate statistical tests utilised following Shapiro-Wilk normality test. P < 0.05 was considered significant. Where data was missing, it was not imputed.

## Results

### Acute effect of glucocorticoids on ICP

**Fig. 1:**
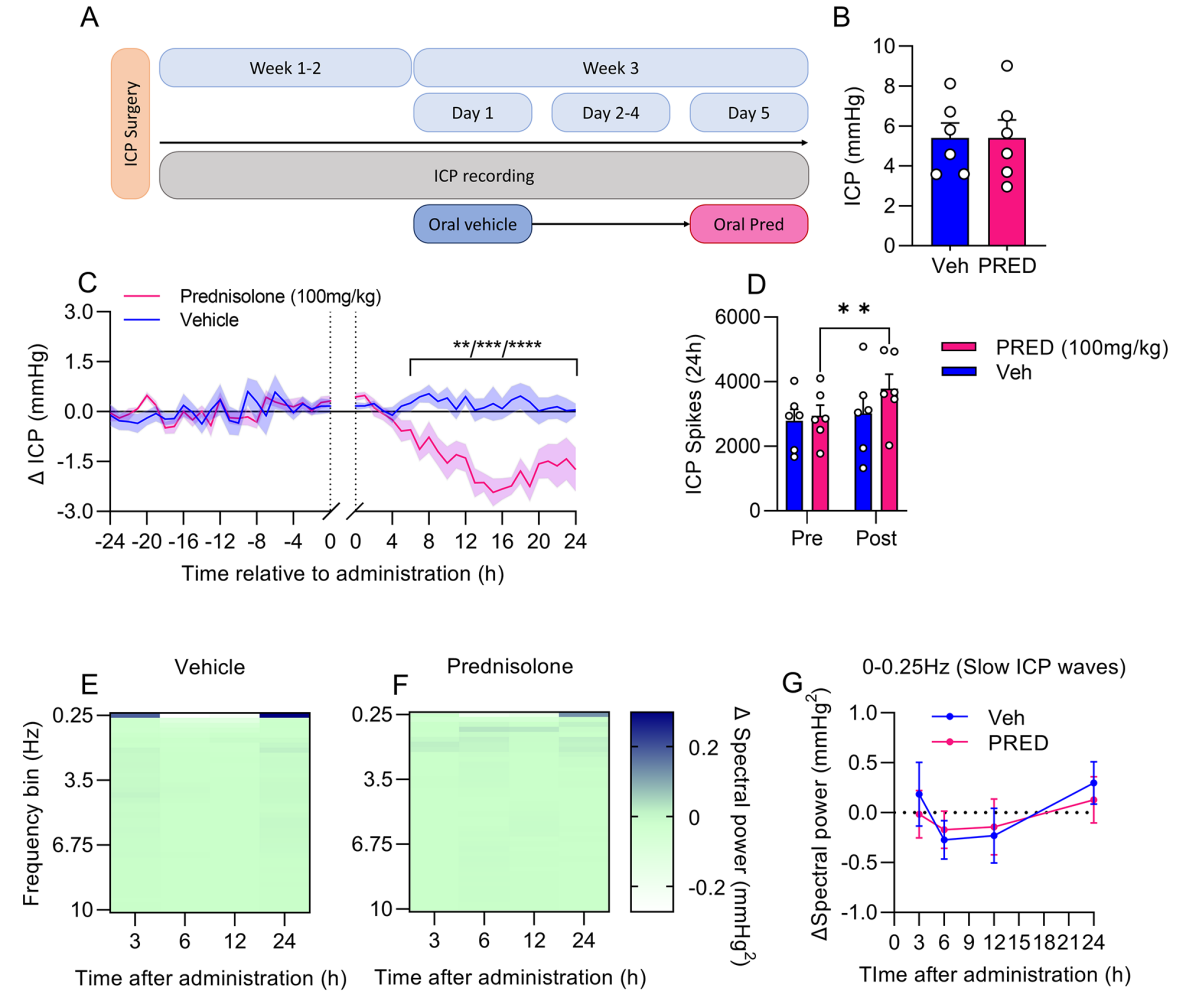
Acute prednisolone reduces ICP Female rats implanted with ICP telemeters treated with either vehicle (0.9% saline) or prednisolone (PRED) (100 mg/kg) administered via oral gavage in a sequential study. **A**) Study design diagram. **B**) Baseline ICP at each treatment for preceding 24 h. **C**) ICP trace displaying 1-hour ICP means 24 h prior to and after oral gavage (graph break), with inset displaying baseline ICP. **D**) ICP spikes in the 24 h prior to and after oral gavage in both vehicle and PRED treated rats. Temporal spectrograms of fast Fourier transformed ICP traces in 0.25 Hz frequency bins of Veh **E**) and PRED **F**) treatment. **G**) Spectral power over 24 h relative to baseline for slow ICP waves (0-0.25 Hz). Two-way repeated measures ANOVA with post-hoc Sidak’s tests for C, D and G. Unpaired t-test for B. N = 6. **=P < 0.01, ***=P < 0.001 and ****=P < 0.0001. Data presented as mean ± SEM and mean for E and F

To determine the effect of a clinically relevant dose of GC on ICP, we administered prednisolone (PRED) (100 mg/kg) via oral gavage in a sequential study (Fig. 1A), which replicates a human pulse dose. ICP was identical at the start of each treatment (5.40 ± 0.74 vs. 5.41 ± 0.89 mmHg, P = 0.97, Fig. 1B). This study was performed when rats had fully recovered from surgery.

One hour after PRED administration ICP inflected downwards, where at 7 h after PRED administration ICP was significantly lower compared to vehicle treatment (0.44 ± 0.17 vs. -1.13 ± 0.35 Δ mmHg, P = 0.0003, Fig. 1C). ICP reduction reached its nadir at a ICP reduction of 48% 15 h after administration (0.24 ± 0.52 vs. -2.43 ± 0.41 Δ mmHg, P < 0.0001, Fig. 1C). From here ICP in PRED treated rats began returning to baseline/vehicle but remained significantly lower than vehicle 24 h after administration (0.05 ± 0.18 vs. -1.75 ± 0.66 Δ mmHg, P < 0.0001, Fig. 1C).

Moreover, we conducted ICP spike analysis. PRED treatment increased the ICP spike frequency by 28.5% (Pre:2943 ± 333 vs. Post: 3783 ± 333 spikes, P = 0.0075, Fig. 1D) over 24 h, whereas vehicle treated rats had unaltered ICP spike frequency (Pre:2788 ± 357 vs. Post: 3017 ± 537 spikes, P = 0.56, Fig. 1D).

To further interrogate the effect of PRED on the ICP phenotype, we conducted ICP waveform analysis (Fig. 1E,F). No temporal differences in ICP waveforms were found with PRED treatment compared to control, including clinically meaningful slow ICP waves (0-0.25 Hz) (Fig. 1G).

### The chronic effect of glucocorticoids on ICP

**Fig. 2:**
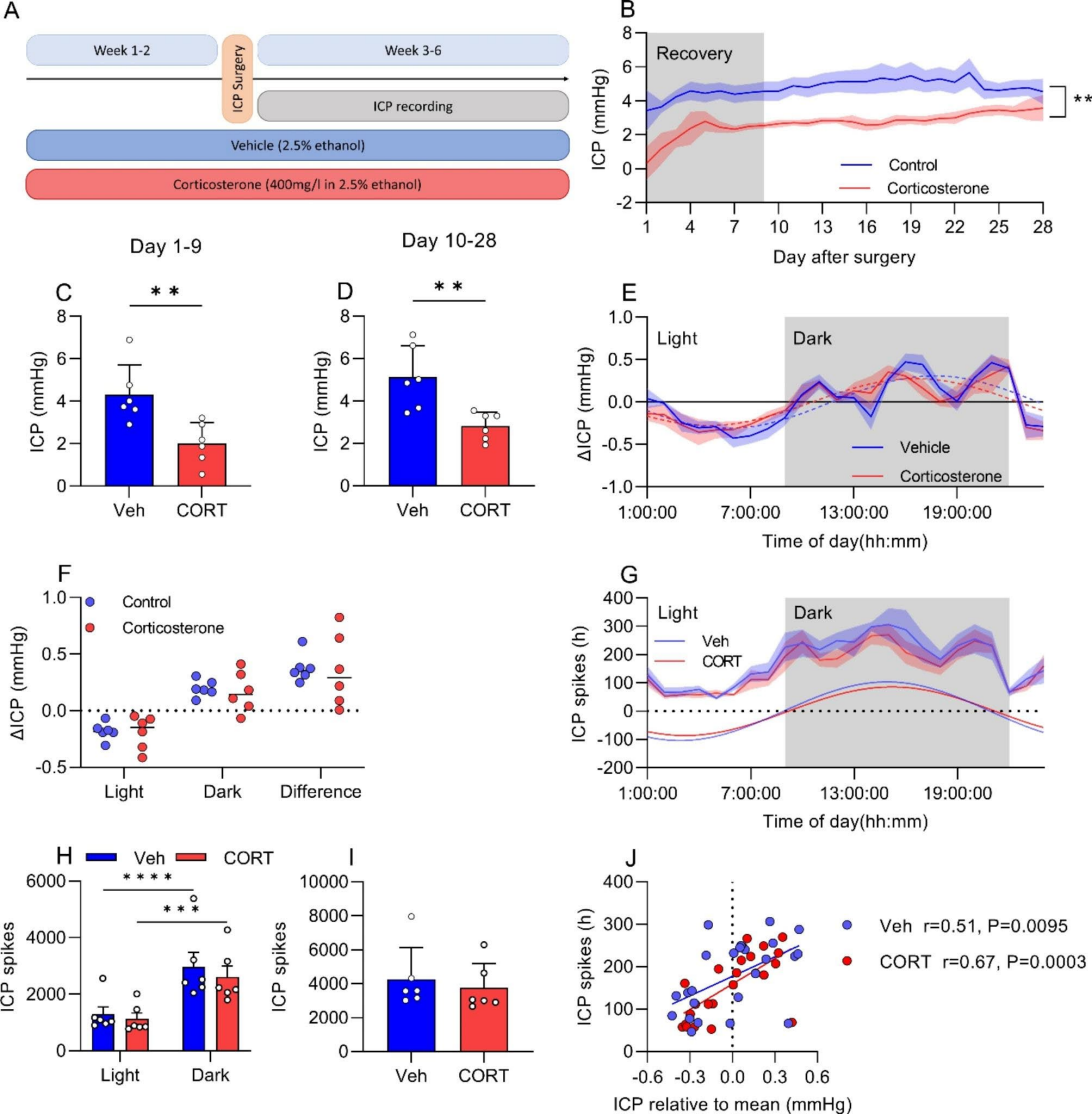
Chronic corticosterone lowers ICP Female rats implanted with ICP telemeters treated with either vehicle (2.5% ethanol) or corticosterone (CORT) (400 mg/L) in their drinking water over 28 days. **A**) Study design diagram. **B**) ICP trace of implanted rats over 28 days displaying whole day ICP means. **C**) Mean ICP from day 0-9 in implanted rats. **D**) Mean ICP from day 10-28 in implanted rats.' **E**) Mean ICP day of 5 days with ICP periodicity lines. **F**) Light dark differences In ICP. **G**) Mean daily ICP spikes, average of 5 days with periodicity lines. **H**) ICP spikes in light and dark, and **I**) total daily spikes. **J**) Scatter graph of hourly ICP spikes vs. ICP relative to mean. Students t-test for C,D and I. Repeated measures ANOVA with Sidak’s test for B, and F. Spearman’s correlation coefficient for I. N = 6 for B-H, N = 24 for J. **=P < 0.01, ***=P < 0.001 and ****=P < 0.0001. Data presented as mean ± SEM, mean for F

To assess the effect of chronic GCs on ICP we pre-treated rats with either exogenous CORT or vehicle water prior to telemeter implantation (Fig. 2A).

Rats treated with exogenous CORT had ICP that was consistently lower over the 4-week implantation period (P = 0.0052, Fig. 2B). In days 1–9 after surgery, when rats physiological parameters including ICP recovery are recovering from surgery [21], we demonstrated that exogenous CORT is associated with lower ICP (4.32 ± 0.57 vs. 2.02 ± 0.40 mmHg, P = 0.0079, Fig. 2C). In days 10–28 after surgery, when rats have fully recovered from surgery, we demonstrate that exogenous CORT is associated with lower ICP by 44% (5.12 ± 0.612 vs. 2.82 ± 0.27 mmHg, P = 0.0064, Fig. 2C).

ICP is well known to display diurnal variation. Here we investigated whether exogenous GCs could affect this. It was found that both vehicle and CORT treated rats display a cosine fit for the average ICP day trace (P < 0.0001), indicating that exogenous CORT did not eliminate the periodicity of ICP over the course of the day (Fig. 2E). Moreover, CORT did not modify the light/dark difference in ICP (Fig. 2F).

Given that we found that PRED affects ICP spiking behavior, we assessed the effect of CORT on ICP spikes. Both vehicle and CORT treated rats fulfilled the criteria for rhythmicity in ICP spikes, having a cosine fit (P < 0.0001) (Fig. 2G) where ICP spikes occurred more frequently in the dark in both vehicle (light:1291 ± 257 vs. dark: 2971 ± 509 spikes, P < 0.0001) and CORT (light: 1139 ± 203 vs. dark:2617 ± 382 spikes, P = 0.0002) treated rats (Fig. 2H). No difference was found in total ICP spikes between vehicle or CORT treated rats (4264 ± 761 vs. 3757 ± 583 spikes, P = 0.6, Fig. 2I). Interestingly, ICP spikes positively correlated with ICP relative to the mean (Fig. 2J). Here, both vehicle (r = 0.51, P = 0.0095) and CORT (r = 0.67, P = 0.0003) treated animals displayed this correlation, where both the intercept (P = 0.51) and the gradient (P = 0.32) were the same, suggesting that CORT does not alter the relationship between ICP spikes and ICP.

**Fig. 3:**
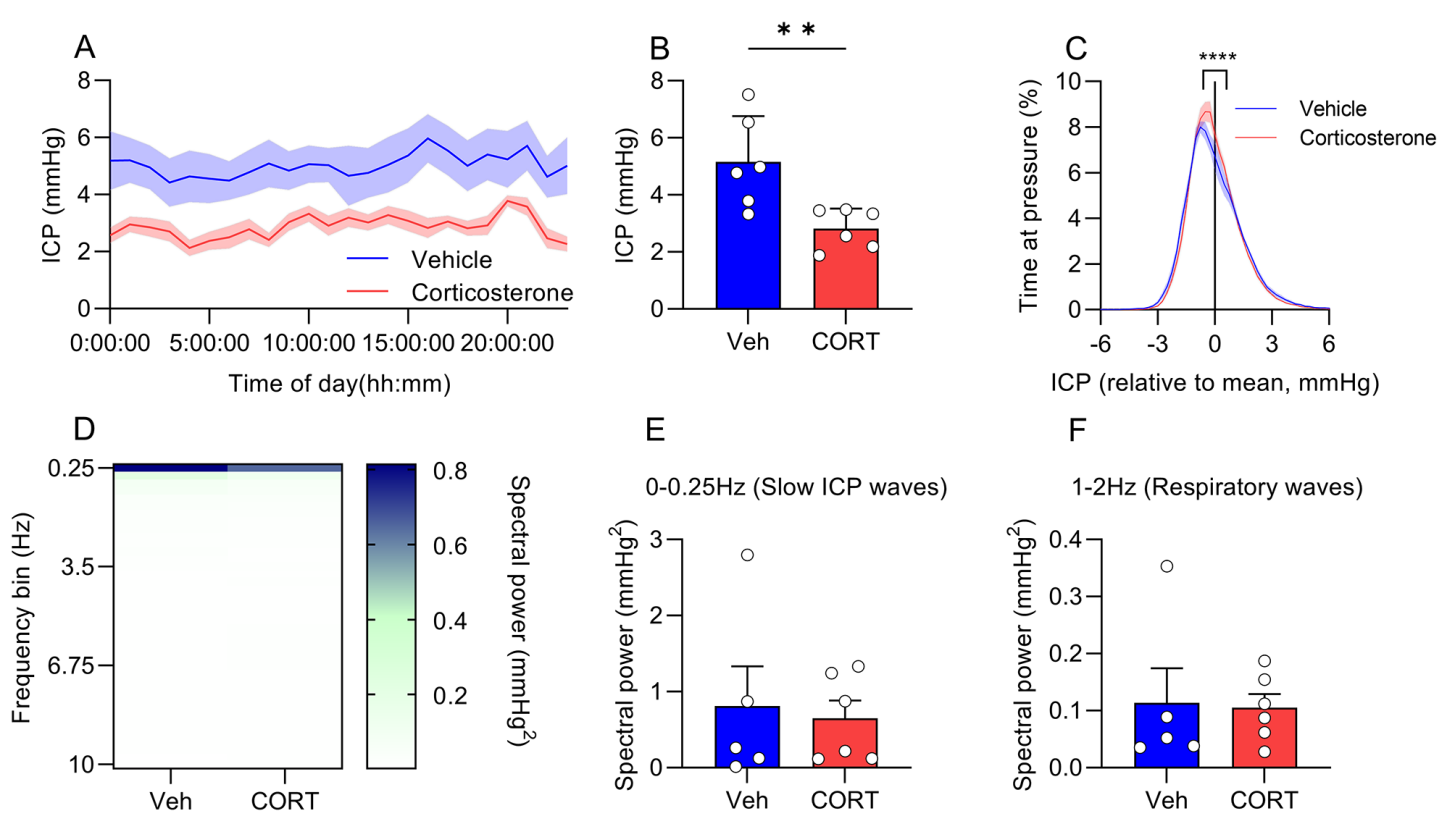
ICP features during chronic administration of corticosterone Female rats implanted with ICP telemeters treated with either vehicle (2.5% ethanol) or CORT (400 mg/L), ICP features on day 20. **A**) ICP trace. **B**) Mean ICP on day 20. **C**) ICP around the mean for each individual animal. **D**) ICP spectrogram of fast Fourier transformed ICP traces in 0.25 Hz frequency bins at 12:00. Spectral power in **E**) 0-0.25 Hz and **F**) 1-2 Hz. Unpaired t-tests for B,E and F. ANOVA with post-hoc Sidak’s tests for C. N = 6. **=P < 0.01, ***=P < 0.001 and ****=P < 0.0001. Data presented as mean ± SEM and mean for D

ICP variance is an important clinical feature, where increased ICP variance is a pathological feature, thus we also analyzed this [25]. On day 20 (Fig. 3A), where ICP was lower in CORT treated rats (5.15 ± 0.65 vs. 2.82 ± 0.28 mmHg, P = 0.0085, Fig. 3B) we conducted the analysis. Here we demonstrated that exogenous CORT increases the percentage of time in the − 0.5 mmHg (7.83 ± 0.39 vs. 8.68 ± 0.42%, P < 0.0001), -0.25 mmHg (7.29 ± 0.38 vs. 8.66 ± 0.47%, P < 0.0001), 0 mmHg (6.72 ± 0.63 vs. 7.67 ± 0.33%, P < 0.0001), 0.25 mmHg (6.11 ± 0.60 vs. 6.90 ± 0.28%, P < 0.0001) and 0.5 mmHg (5.49 ± 0.50 vs. 6.26 ± 0.22%, P = 0.0001) bins, relative to the mean (Fig. 3C). This equates to a 4.73% increase, or 68 more minutes a day spent at ICP − 0.5 to 0.5 mmHg around the mean for CORT treated rats.

To further interrogate the ICP phenotype we conducted an ICP waveform analysis on day 20 (Fig. 3D). No differences were found between vehicle and CORT treated rats in physiologically meaningful frequency bins, 0-0.25 Hz (slow ICP waves) (Fig. 3E) and 1-2 Hz (respiratory ICP waves) (Fig. 3F).

#### Gene expression at choroid plexus

**Fig. 4:**
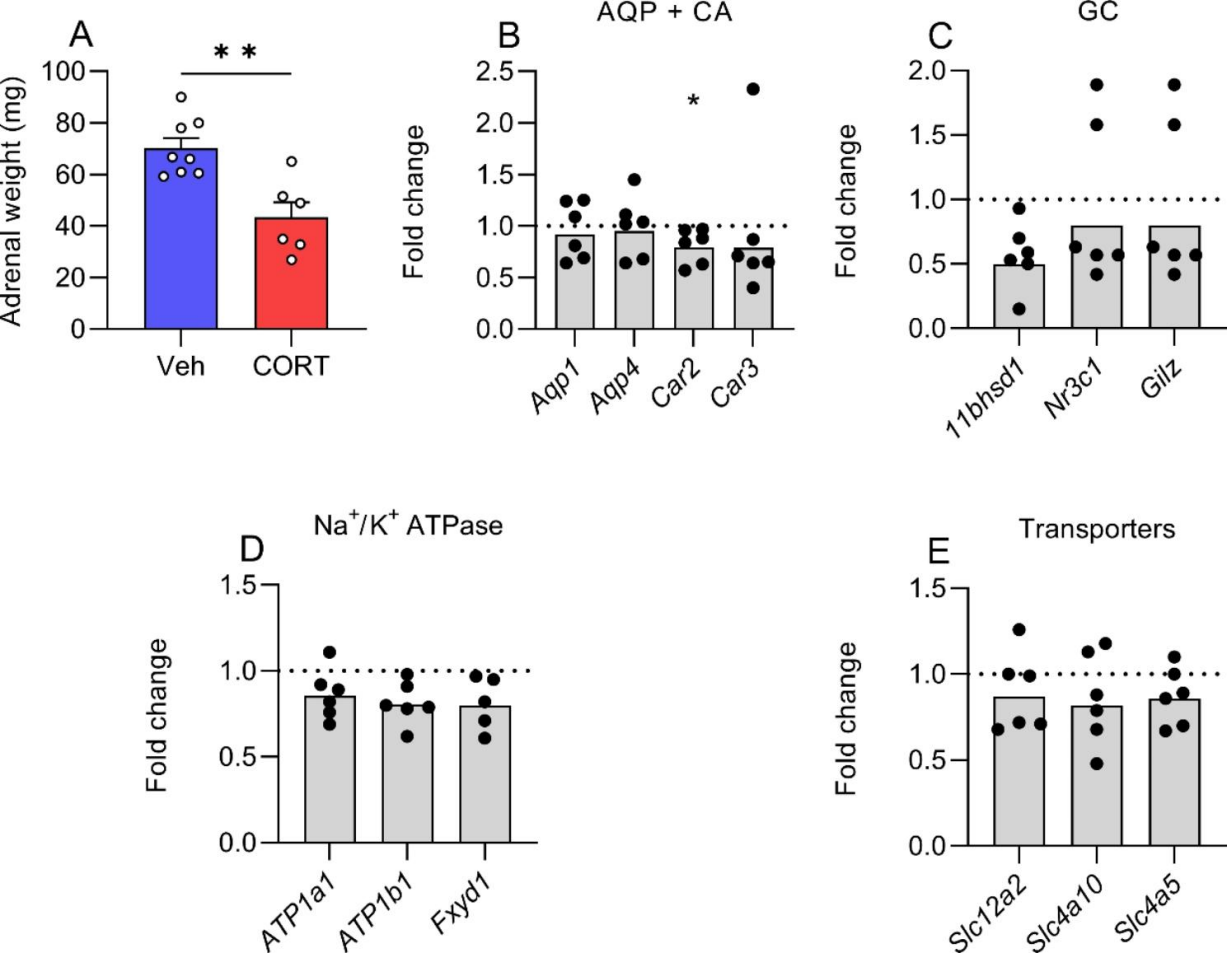
The effect of corticosterone on CP gene expression Female rats implanted with ICP telemeters treated with either vehicle (2.5% ethanol) or CORT (400 mg/L) for 6 weeks. **A**) Adrenal gland weights. Fold change gene in choroid plexus of **B**) *Aqp1, Aqp4, Car2, Car3*, **C**) *11bhsd1, Nr3c1, Tsc22d3*, **D**) *Atp1a1, Atp1b1, Fxyd1.***E**) *Slc4a10, Slc4a5 and Slc12a2.* Statistics performed on ΔCt values. Unpaired t-test for all. N = 8 for vehicle and N = 6 for CORT. *=P < 0.05 and **=P < 0.01. Data presented as mean ± SEM for A and as geometric mean for B-E

Given that exogenous CORT modifies ICP, we investigated the effects of CORT on the expression of genes associated with CSF secretion at the CP, the organ responsible for the majority of CSF secretion. Here, CORT treated rats had smaller adrenal glands (70.18 ± 3.96 vs. 43.37 ± 5.82 mg, P = 0.0019, Fig. 4A) than vehicle treated rats, indicating long term GC excess. Rats had received treatment for a total of 6 weeks at time of tissue collection. We found that exogenous CORT reduced the expression of *Car2* by 0.82-fold (0.52 ± 0.08 vs. 0.86 ± 0.15 ΔCt, P = 0.047, Fig. 4B), encoding for carbonic anhydrase 2, although we found no changes in the expression of *Aqp1*, *Aqp4* and *Car3 (*Fig. 4B). Moreover, we found no alteration in the genes associated with GC action namely *11bhsd1*, *Nr3c1* (GC receptor) and *Tsc22d3* (*Gilz*) (Fig. 4C). Furthermore, no difference in the expression of *Atp1a1*, *Atp1b1* and *Fxyd1*, Na^+^/K^+^ ATPase components were found (Fig. 4D). We did not find altered expression in *Slc4a10*, *Slc4a5* and *Slc12a2*, whose activity is fundamental in CSF secretion (Fig. 4E). Ct values can be found in supplemental Table 1.

## Discussion

GCs are among the most prescribed drugs, and endogenously are essential for life. Among their clinical uses, they are used to treat pathologically raised ICP. Although the functional rationale is clear for treating cerebral oedemic processes, it is unclear if GCs modify ICP during normal cerebral physiology. Here we assessed the effects of acute PRED and chronic exogenous CORT on ICP in freely moving rats.

We demonstrated that acute treatment with high dose oral PRED, equivalent to a human pulse dose, substantially reduced ICP by 48%. This effect was induced rapidly and was sustained for at least 17 h and did not return to baseline during the 24-hour recording period. Assessing when ICP returns to baseline after acute treatment will be of interest in future studies. Moreover, we provided evidence that chronic exogenous CORT reduced ICP by 44%, where ICP is lower throughout the entire ICP monitoring period. ICP was already lower after the 2-week pretreatment and given the present data, reduced ICP could be considered another side effect of GC therapy.

We found that acute PRED and chronic CORT lower ICP to a similar, substantial degree of 48% and 44% respectively, corroborating previous experimental data suggesting that GCs can reduce ICP in bacterial meningitis in rabbits [11]. This compares favorably to other drugs that reduce ICP. Glucagon-like peptide-1 receptor agonists have been demonstrated to reduce ICP in female rats by 40% both acutely and chronically [26]. Additionally, classical ICP lowering drugs, acetazolamide and topiramate, reduce ICP by 10–12% at clinically meaningful doses [27, 28]. The present study failed to assess the role GCs have on non-oedemic raised ICP, such as diet induced obesity (DIO) [29]. We demonstrated that GCs are efficacious in reducing ICP, thus GC preparations may be suitable in the clinic for chronic ICP reduction therapy, however the broad side effect profile of GCs should be taken into consideration prior to commencing any treatment. Future experiments using different doses and other GR agonists, including GC prodrugs, those that can be metabolized by 11β-HSD1 to active GCs, should be performed.

The disease IIH is associated with raised ICP where GCs have been proposed to be pathophysiologically relevant to the raised ICP [29]. It has been demonstrated that IIH patients have raised systemic GC as measured in the urine [17]. Moreover, multiple studies have demonstrated that reduction of ICP is associated with reduction in endogenous active GCs, suggesting a role for GCs in the raised ICP pathology [13, 17, 30]. Indeed, the 11β-HSD1 inhibitor AZD4017 has been previously demonstrated to reduce ICP in IIH by 4.3 cmH_2_O, a 22% reduction [30]. These data are in contrast to the present study and the bulk of the literature where GCs are reported to reduce ICP in pathological states. IIH is a complex metabolic disease treatable by weight loss, where GC excess is associated with metabolic dysfunction [31, 32]. Given these, it could be speculated that the GC phenotype in IIH is a symptom of the underlying metabolic disease with no relevant role in the ICP pathophysiology. Consequently 11β-HSD1 inhibition, which modifies systemic metabolism in IIH beneficially, could then modify ICP indirectly rather than a direct effect of GCs raising ICP [33]. Future studies assessing the direct effect of 11β-HSD1 inhibitors on ICP are warranted.

We provide for the first-time evidence that ICP spikes display diurnal variation, where ICP spikes occur more frequently during the active (dark) period for rats. Exogenous CORT did not modify spike periodicity or ICP spike frequency. Increased ICP spike frequency is associated with pathological situations such as stroke in rats [34]. Consequently, it is interesting that we observed an increase in ICP spikes with oral PRED, in contrast to CORT. PRED is a more specific GR agonist, whereas CORT is the endogenous rodent GC and agonizes both GR and mineralocorticoid receptor (MR) to a similar degree [35]. As such any difference in their effects could be due to different sites and mechanism of action. Given the different potencies of PRED and CORT at the GR, a higher degree of GR activation could be required to alter ICP spikes.

Endogenous GCs have a strong diurnal component, which dictates multiple physiological processes [4]. However, exogenous CORT did not alter the diurnal pattern of ICP. This is despite our CORT dose inducing adrenal atrophy, demonstrating the dose is sufficient to replace endogenous GC output. Thus, the data may suggest diurnal variation in ICP is not driven by GC rhythmicity. This study provides evidence that exogenous CORT reduces ICP variation around the mean. This could represent either an alteration in cerebral pressure dynamics or indicate that a biological minimum in ICP has been reached. It has been previously demonstrated that DIO, a condition associated with tissue specific GC dysfunction, does not alter ICP variation, as such it is interesting that CORT in of itself can alter ICP variation [29].

We have previously demonstrated that when socially isolated, a stressful state that increases circulating CORT, rats have lower ICP compared to rats that are co-housed [21, 36]. Our present results could explain this phenomenon, stress induced GC increases could lower ICP. This highlights the need to minimize environmental stress to prevent stress responses confounding ICP data.

The mechanisms underlying the ICP reduction found in this study remains unelucidated. Although I.V dexamethasone and methylprednisolone, and oral betamethasone have been demonstrated to reduce CSF secretion, more recent evidence describes that intraventricularly administered hydrocortisone increases CSF secretion [15, 37, 38]. Such differences could be explained through administration route or species differences. Physiological maintenance could also explain the differences in results: those studies that ventilated their subjects, and thus maintained normal physiological parameters, reported reductions in CSF secretion with GCs, whereas the study without ventilation reported increased CSF secretion with GCs [15, 37, 38]. Extra-choroidal sources of CSF generation, such as the parenchyma and cerebral vasculature, may also have their properties altered by GCs [39].

We observed minor changes in the gene expression associated with CSF secretory genes at CP with chronic exogenous CORT, only observing a reduction in *Car2* expression. *Car2* encodes for carbonic anhydrase 2, where inhibition of carbonic anhydrase 2 has been demonstrated to reduce CSF secretion and ICP [28, 40]. These results are congruent with the suggestion that GCs lower CSF secretion. The results in CP are minor compared to the broad effects GCs have ICP and on tissues such as the liver and adipose [6]. The CP is a GC target tissue, expressing functional 11β-HSD1 in multiple species and GCs have been demonstrated to have a direct effect on CP CLOCK gene activity in rats [12–14]. We would expect to find the GC response gene *Gilz* to have raised expression in CORT treated rats. However, the sampling of tissue at the transition from light to dark could mean that the rats may not have raised systemic CORT due to reduced drinking during light (resting) hours, limiting the transcriptomic effects.

ICP is also determined by CSF drainage. Previous studies have demonstrated that acute and chronic GCs do not modify CSF drainage rate [15, 16]. Of clinical interest, GC withdrawal decreases CSF drainage, without a concurrent ICP increase in dogs [16]. Further work is required to elucidate how GCs modulate ICP and CSF dynamics.

## Limitations

Although we demonstrated a GR specific agonist PRED, reduces ICP, we cannot exclude the role of mineralocorticoids also modulating ICP given that CORT is a physiological agonist of both MR and GR [41]. Future experiments utilising dexamethasone and fludrocortisone, GR and MR agonists respectively, could differentiate between exclusive GC and MR effects on ICP.

The vehicle treated rats received 2.5% ethanol in the chronic study, a relatively high dose, where the effects of ethanol on ICP are unknown. However, the mean ICP of 5mmHg in the vehicle treated rats is in keeping with previous literature for normal ICP in female rats [21, 29]. Consequently, any effect of ethanol on ICP is likely small. Future assessment of the effects of ethanol on ICP is warranted.

This study used exclusively female rats due to the potential pathophysiological link to IIH that almost exclusively affects young females. This limits applicability of the present results to females only. Future studies on male rats are required to confirm the results of this study. We did not design the study to observe the initial onset of ICP reduction with CORT. Given that this would be of clinical interest future assessment of the dynamics of the initial effects of CORT on ICP are warranted. Given the lack of relevant transcriptomic changes at the CP, protein level and functional changes should be assessed in the future.

Withdrawal of GCs has been reported to induce an increase in ICP in several human case studies [42, 43]. Although this is an interesting clinical phenomenon, the present study was not designed to assess this rare clinical feature. Future studies should assess the role of GC withdrawal on ICP.

## Conclusions

Here we provide evidence that both acute and chronic GC monotherapy can reduce ICP in normal pressure female rats. Moreover, in the chronic setting the ICP reduction was sustained with no tachyphylaxis or indication of pathological features. Thus, we provide preclinical evidence endorsing the use of GCs to treat raised ICP in the acute setting. GC therapy may have utility to treat raised ICP chronically.

## Electronic supplementary material

Below is the link to the electronic supplementary material.

Supplementary Material 1

## Data Availability

Data are available upon reasonable request.
