# Supplementary material for: Glucocorticoids modify intracranial pressure in freely moving rats
Source: Fluids Barriers CNS. 2023 May 25;20:35. doi: 10.1186/s12987-023-00439-y (PMC10214560; doi:10.1186/s12987-023-00439-y)
Supplement: Supplementary file 1 — Supplementary Material 1 [file 12987_2023_439_MOESM1_ESM.docx]

| Gene | Vehicle (n=8) | Corticosterone (n=6) |
| --- | --- | --- |
| *Aqp1* | 4.008±0.199 | 4.131±0.174 |
| *Aqp4* | 7.279±0.309 | 7.597±0.183 |
| *Car2* | 0.375±0.048 | 0.711±0.132* |
| *Car3* | 9.104±0.397 | 9.825±0.348 |
| *Slc12a2* | 1.248±0.085 | 1.447±0.147 |
| *Slc4a10* | 2.068±0.149 | 2.356±0.198 |
| *Slc4a5* | 0.931±0.099 | 1.138±0.113 |
| *ATP1a1* | 2.026±0.133 | 2.251±0.098 |
| *ATP1b1* | -0.874±0.099 | -0.581±0.089 |
| *Fydx1* | 0.383±0.181 | 0.478±0.250 |
| *11bhsd1* | 7.873±0.331 | 8.881±0.376 |
| *Nr3c1* | 6.819±0.276 | 7.146±0.362 |
| *Tsc22d3* | 7.552±0.303 | 8.137±0.171 |

**Supplemental table 1**. 6-week GC or vehicle treatment, relating to figure 4.

ΔCt values of genes assessed in choroid plexus from treated rats. Students t-test. Mean±SEM. *=P<0.05. N in parentheses.
